# Draft Genome Sequences of 10 *Bacillus subtilis* Strains That Form Spores with High or Low Heat Resistance

**DOI:** 10.1128/genomeA.00124-16

**Published:** 2016-03-17

**Authors:** Erwin M. Berendsen, Marjon H. J. Wells-Bennik, Antonina O. Krawczyk, Anne de Jong, Auke van Heel, Robyn T. Eijlander, Oscar P. Kuipers

**Affiliations:** a Molecular Genetics, University of Groningen, Groningen, Groningen, The Netherlands; b NIZO food research, Ede, The Netherlands; c Top Institute Food and Nutrition (TIFN), Wageningen, The Netherlands

## Abstract

Here, we report the draft genome sequences of 10 isolates of *Bacillus subtilis*, a spore forming Gram-positive bacterium. The strains were selected from food products and produced spores with either high or low heat resistance.

## GENOME ANNOUNCEMENT

*Bacillus subtilis* is a soil-dwelling organism that can also be found in the mammalian microbiota (1). *B. subtilis* strains can be used as cell factories for enzyme or metabolite production, as biocontrol agents, or even as probiotics (2). However, they can also form low- or high-heat-resistant spores that may survive food processing techniques and cause food spoilage in consumer products. Ten strains of *B. subtilis* were isolated as spores from food products (Table 1). The spore heat resistance traits of some of these strains were described in a study by Berendsen et al. (3). The sequences of these strains will provide valuable information on genes involved in sporulation and germination (4, 5). Moreover, these and previously published *B. subtilis* strains can be grouped according to phenotype, and subsequent gene trait matching can be used to identify genes involved in the phenotype of interest.

**TABLE 1:** Genome features and GenBank accession numbers of the strains

Strain ID	Strain	Isolate source	Accession no.	Genome size (Mb)	Coverage (×)
B4067	*B. subtilis* A163	Chicken soup	JSXS00000000	4.31	1,168
B4068	*B. subtilis* CC2	Curry cream	JXHK00000000	3.98	227
B4069	*B. subtilis* IIC14	Binding flour	JXHL00000000	4.09	434
B4070	*B. subtilis* A162	Peanut chicken soup	JXHM00000000	4.28	237
B4071	*B. subtilis* CC16	Curry cream soup	JXHN00000000	4.2	323
B4072	*B. subtilis* RL45	Red lasagna sauce	JXHO00000000	4.09	197
B4073	*B. subtilis* MC85	Curry soup	JXHP00000000	4.13	285
B4143	*B. subtilis*	Surimi	JXLQ00000000	4.2	311
B4145	*B. subtilis*	Cereals	JXHQ00000000	4.4	258
B4146	*B. subtilis*	Mayonnaise	JXHR00000000	4.26	313

The 10 strains were grown overnight in 10 ml of brain heart infusion (BHI) broth (Difco) at 37°C and harvested at the exponential-growth phase. After centrifugation, the cell pellet was resuspended in SET buffer (75 mM NaCl, 25 mM EDTA, 20 mM Tris-HCl [pH 7.5]) and incubated with lysozyme (2 mg/ml) and RNase (0.4 mg/ml) for 30 min at 37°C. Subsequently, the sample was treated with SDS (1% final concentration) and proteinase K (0.5 mg/ml) at 55°C for 60 min. Genomic DNA was extracted from the lysate with phenol-chloroform, precipitated with isopropanol and sodium acetate (300 mM), and dissolved in Tris-EDTA (TE) buffer. The isolated DNA was sheared to 500-bp fragments in the Covaris (KBiosciences) ultrasonic device for preparing the next-generation sequencing (NGS) library preps using the paired-end NEB NextGen library preparation kit. The libraries were 101-base paired-end sequenced on an Illumina HiSeq 2000 by multiplexing 12 samples per flow cell. Velvet (6) was used to perform a *de novo* paired-end assembly on each of the 10 genomes, resulting in the draft genome sequences (Table 1). Annotation of the genomes was done using the following steps: (i) scaffolds were uploaded to the RAST server (7) and automatically annotated using the SEED bases method on this server, (ii) the resulting annotated scaffolds were mapped using CONTIGuator (8) on their closest neighbor (identified by RAST) to generate the pseudogenome, (iii) locus tags were added to each feature using an in-house-developed Perl script, according to the NCBI standard, (iv) BAGEL3 (9) was used to find and annotate bacteriocin gene clusters, and (v) the protein annotation was extended using InterProScan (10).

### Nucleotide sequence accession numbers.

The genome sequence of the 10 *B. subtilis* strains have been deposited as whole-genome shotgun projects at DDBJ/EMBL/GenBank under the accession numbers listed in Table 1.
